# Finding Near-Optimal Groups of Epidemic Spreaders in a Complex Network

**DOI:** 10.1371/journal.pone.0090303

**Published:** 2014-04-02

**Authors:** Geoffrey Moores, Paulo Shakarian, Brian Macdonald, Nicholas Howard

**Affiliations:** 1 Electrical Engineering and Computer Science Department, United States Military Academy, West Point, New York, United States of America; 2 Mathematical Science Department, United States Military Academy, West Point, New York, United States of America; 3 Network Science Center, United States Military Academy, West Point, New York, United States of America; Universidad de Zarazoga, Spain

## Abstract

In this paper, we present algorithms to find near-optimal sets of epidemic spreaders in complex networks. We extend the notion of local-centrality, a centrality measure previously shown to correspond with a node's ability to spread an epidemic, to sets of nodes by introducing combinatorial local centrality. Though we prove that finding a set of nodes that maximizes this new measure is NP-hard, good approximations are available. We show that a strictly greedy approach obtains the best approximation ratio unless P = NP and then formulate a modified version of this approach that leverages qualities of the network to achieve a faster runtime while maintaining this theoretical guarantee. We perform an experimental evaluation on samples from several different network structures which demonstrate that our algorithm maximizes combinatorial local centrality and consistently chooses the most effective set of nodes to spread infection under the SIR model, relative to selecting the top nodes using many common centrality measures. We also demonstrate that the optimized algorithm we develop scales effectively.

## Introduction

In this paper we look to find *optimal* sets of individuals in a complex network to initiate an epidemic. Addressing such a problem will have clear implication in seeding a social network to ensure a given phenomenon diffuses optimally and may also provide insight into mitigation strategies against an infection initiated by a group of individuals. Further, this problem is non-trivial. For instance, it has previously been noted in [1] that selecting a second influential node, or ‘spreader,’ does not always significantly increase the spread of the epidemic. In [2], the authors show that identifying an optimal set of spreaders under a more generalized epidemic model is NP-hard.

The susceptible-infected-recovered (SIR) model [3] is one of the most well-studied models of epidemic disease spread in a population. In this model, individuals in a population are in one of three states: *susceptible* individuals can acquire the disease from *infected* individuals who after a certain amount of time become *recovered* and can no longer transmit or acquire the disease. In recent studies, there has been much interest in studying this model on populations structured as a network [4]–[6].

Throughout this paper, we will assume a population structured as an undirected network 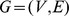 where 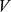 is a set of individuals (“nodes”) and 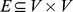 where 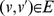 implies 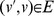. The size of 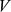 and 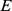 are denoted 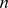 and 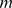 respectively. For other sets of elements, we shall use the notation 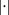 to denote the size of that set. For a given node 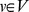, 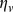 is the set of neighbors, formally 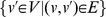. We will extend this to sets: for set 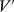, 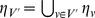. We will use 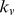 to denote the degree of 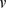 which is the cardinality of 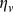. The we denote the average and maximum degree of any node in the graph as 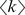 and 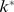 respectively. We note that, in most real-world social networks, 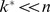. The quantity 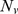 is the number of neighbors and next-nearest neighbors of node 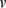 and is defined formally as follows:
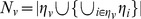
(1)


In this paper, we use a the version of the SIR model specified by Chen et al. [7]. Nodes in the network under this version of the model are in one of three states: susceptible, infected, or recovered. Once a node is infected, one of its neighbors then becomes infected at random (by a uniform probability over the neighbors of the initially infected node). After infecting a neighbor, the node then recovers with a probability 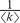.

Chen et al. accurately identified individual spreaders with Local Centrality. For a given node 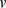, its Local Centrality, 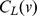 is defined as follows:
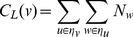
(2)


We extend Local Centrality with a set centrality-based technique similar to that of [8], [9]. We then frame an optimization problem that seeks to find 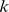 of nodes in the network that together optimize our extended version of local-centrality. For some set 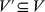 with *combinatorial local centrality*, denoted 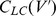, is defined as follows:
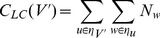
(3)


**Figure 1:**
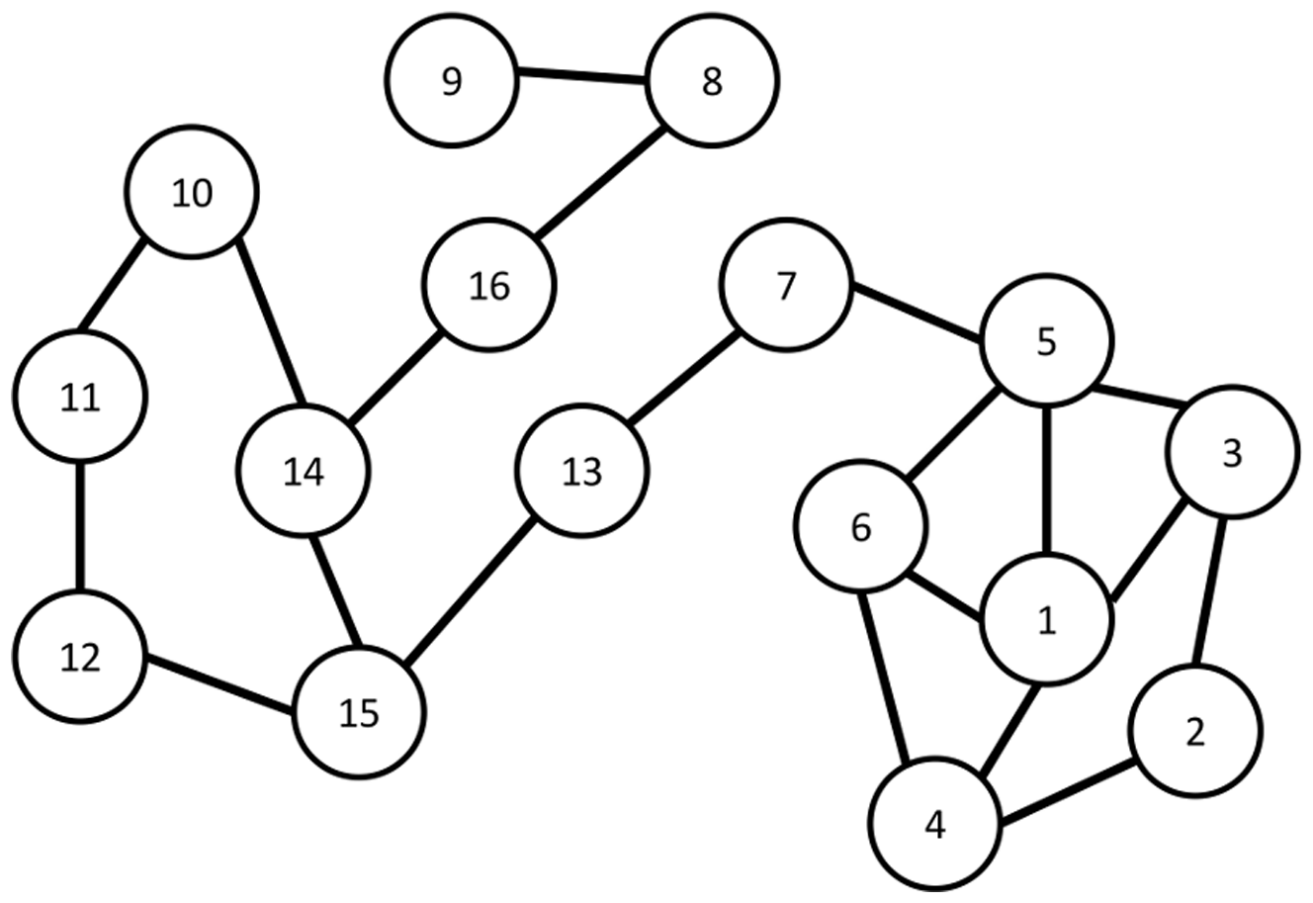
An Example Network. 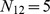
, 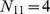, and 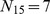. 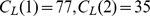, and 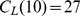. 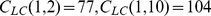. Note that although the local-centrality for node 2 is higher than that of 10, the combinatorial local centrality is higher when nodes 1 and 10 are paired, rather than 1 and 2.

Figure 1 demonstrates 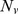, 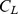, and 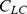 on a small, arbitrary network.

Using this definition, we now present the problems we wish to study in this paper which deal with finding a set of nodes (of sized 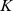) that optimizes the above function.

### 

#### Definition 1

*Max Combinatorial Local Centrality Problem (Max* 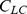 *):*

*INPUT*: 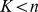;

*OUTPUT*: 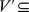 *V*, *s.t.* 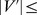 *K and* 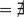 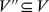 *s.t.* 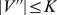 *where* 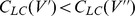


#### Definition 2

*Combinatorial Local Centrality Decision Problem (Dec* 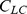 *)*

*INPUT*: 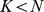; 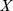


*OUTPUT*: *Yes if* 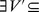 *V, s.t*. 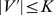 *and* 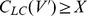; *No otherwise*.

Unfortunately, the MAX 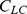 problem is also NP-hard and difficult to approximate as well. However, we demonstrate certain mathematical properties of the problem (namely sub-modularity and monotonicity) that allow us to leverage the results of [10] to prove that a greedy approach achieves the best approximation ratio unless P = NP. We then create an algorithm that selects nodes in a manner equivalent to the greedy approach but does so more efficiently, hence running faster. This second algorithm maintains the theoretical guarantees of the greedy approach with respect to approximation and improves upon the theoretical guarantees of the greedy approach with respect to runtime.

Both algorithms are then experimentally evaluated to demonstrate a significant speedup of several orders of magnitude with the improved algorithm. We then analyze the experimental spreading potential of a set of vertices chosen with our algorithm against the top 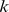 nodes based on several common centrality measures from the literature. We found our GREEDY-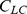 algorithm identifies sets of nodes whose corresponding 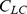 value is consistently greater than that found using centrality measures (average increase as compared to centrality measures was 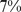). We also compare our approach to the centrality measures based on the expected number of infectees in the aforementioned SIR model. On average, GREEDY-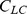 outperforms the other centrality measures (average of 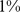). Additionally, we also found that both in terms of optimizing 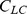 and expected number of infectees, GREEDY-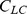 more consistently picked the well-performing sets of nodes than any single centrality measure.

After we review related work, the rest of the paper is outlined as follows. In Algorithms and Analysis, we present the complexity and approximation results for the MAX-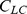 and DEC-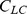 problems. We then show that the a greedy approach obtains the best possible approximation ratio under currently-accepted theoretical assumptions. We then refine our greedy algorithm and produce the theoretical speed-up. Next, our data sets and experimental set-up are provided, followed by our experimental results. We conclude with a brief discussion including directions for future research. Full proofs are contained in the supplemental information section.

### Related Work

Identifying epidemic spreaders in a social network is a very active area of research. For instance, identifying a single node with the ability to spread an epidemic effectively has been previously studied in [1], [5]–[7], [11]. This paper focuses on a different problem: identifying a set of nodes that can optimally spread an epidemic. We build on the centrality measure and epidemic model of [7]. In that work, the authors introduce Local Centrality as a centrality measure which is a trade off between computational complexity and influence prediction, finding a middle ground between measures such as betweenness and degree (respectively too computationally expensive and of little relevance on large networks).

Identifying sets of epidemic spreaders from a combinatorial centrality measurement (similar to what is done in this paper) is discussed in [8] where the authors elegantly discuss the issues with choosing a set of nodes which either promote or disrupt spreading (KPP-POS and KPP-NEG). They also find that off the shelf centrality measures are not well suited to finding such sets. They describe their own greedy algorithm to find sets of nodes using their proposed group centrality measures [9]. approaches KPP-POS and KPP-NEG with an information theory entropy measure and demonstrate positive results in their simulating environment, however the authors note the entropy calculation is too computationally expensive for large networks.

A more generalized epidemic-like model, the *independent cascade* (IC) model was introduced in classic work of [2] and later improved upon in [12] in terms of efficiency (the original work of [2] had scalability issues due to its dependence on simulation runs). However, this framework is somewhat different from the epidemic model introduced in [7] as under the IC model, an infected node has only one chance to spread a contagion before recovering where here the infected node recovers probabilistically. Further, we note that [12] uses a path-based approach where here we use a neighborhood-based approach (which in our tests outperforms the related path-based approach of closeness). Developing a combinatorial path-based heuristic for the model of [7] and comparing it to the algorithm presented in this paper is an important direction for future work.

In [13] they instead focus on a rumor spreading model for social contagion and information propagation, which is similar to the SIR Model but includes a dampening effect where nodes are more likely to become a stifler (similar to recovered nodes) if they are in contact with other spreaders (infectees) or stiflers. They find that k-core index does not determine the spreading capabilities of the nodes but rather whether or not a given node prevents the diffusion of a rumor to a system-wide scale. Additionally [14], and [2] investigate spreading conditions under a linear threshold model, where the activity of neighbor nodes activates currently inactive nodes [14]. finds a formula for the average size of activated nodes given the size of the seed set and note that the existence of cascades are extremely sensitive to small initial sets of active nodes. The dynamics of these models provide rich new testing grounds for our algorithm in future work. We believe the linear threshold model could be particularly conducive to 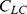 because it tends to avoid clustering in lieu of a more even spread which may result in more areas with inactive nodes surrounded by active nodes. The rumor dynamics model also is disadvantageous for highly clustered infected sets so we may also see positive results under that model.

## Materials and Methods

### Algorithms and Analysis

Here, we present theoretical results on the 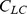 problems defined in the introduction as well as establish algorithms that obtain certain guarantees. First, we examine the computational complexity of the optimization and decision problems associated with maximizing combinatorial local centrality. Unfortunately, these problems are intractable by an embedding on the Max-K-Cover problem of [15] which has previously been proved to be NP-hard.

#### Theorem 1

*The Max* 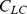 *Problem is NP-Hard.*

#### Theorem 2

*The Dec* 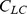 *Problem is NP-Complete*

We will use the notion of approximation introduced in [16] to analyze the performance of our algorithms. Specifically, we define an 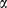-approximate algorithm as follows. Let 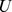 be a universe of elements and 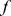 be a function that maps subsets of 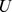 to real numbers. Let 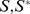 be subsets of 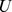 and 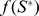 obtains an optimal value and 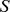 be a subset returned by approximation algorithm 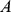. We say that 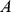 is an 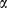-approximate algorithm if 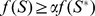. Based on this notion, we are able to leverage another result of [15] to make the following statement on the limit of our ability to approximate Max 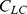 (in polynomial time) under accepted theoretical assumptions.

#### Theorem 3

*Max* 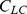 *cannot be approximated in polynomial time within* 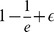 *for* 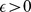 *unless P = NP.*

Knowing this limit, it is desirable to seek an algorithm that obtains a matching approximation ratio. Clearly, such an algorithm would then obtain the best provable approximation unless P = NP, a currently-accepted assumption in computer science. In order to provide such a result, we prove a few important lemmas that we shall require that deal with properties of the function 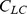. First, we show that it is monotonic. Given set 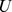, a function 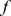 is monotonic iff for any pair of subsets 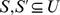 where 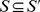, we have 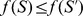.

#### Lemma 1


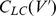
 *is monotonic.*

The next important property we prove about 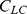 is that it is *sub-modular*. We say a function 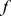 is sub-modular iff for 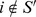 and 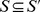, we have 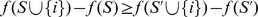.

#### Theorem 4


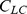
 *is sub-modular.*

Using the properties of monotonicity, we are able to show that a greedy algorithm for approximating 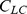 obtains the best approximation ratio unless P = NP. This follows directly from the results of [23]. We include a basic greedy algorithm (GREEDY-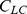, show in in Table 1) and a theorem showing it can run in polynomial time below.

**Table 1:** Algorithm: GREEDY-*C_LC_*.

Algorithm: GREEDY-*C_LC_*	
INPUT: *K*<*n*	
OUTPUT: *V*′ ⊆ *V*, s.t. |*V*′|≤*K* and *V*′ is the greedy solution to Max *C_LC_*.	
1:	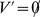
2:	bestVal,curVal = 0
3:	bestInd = null
4:	**while** |*V*′|<*K* **do**
5:	**for** *i*∈*U*−*V*′ **do**
6:	curVal = *C_LC_*(*V*′ ∪ {*i*})−*C_LC_*(*V*′)
7:	**if** curVal>bestVal **then**
8:	bestVal = curVal
9:	bestInd = i
10:	**end if**
11:	**end for**
12:	*V*′ = *V*′∪ {bestInd}
13:	**end while**
14:	RETURN *V*′

#### Theorem 5

*GREEDY-* 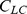 *takes* 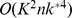 *time.*

The following theorem leverages our two previously described lemmas as well as the construction used in the proof of Theorem 1 to show that the algorithm obtains the best approximation ratio unless P = NP.

#### Theorem 6

*GREEDY-* 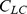 *obtains the best possible approximation ratio in polynomial time unless P = NP*

Though polynomial, the result of Theorem 5 is likely problematic for larger networks. As such is the case we sought to improve upon this run-time with an improved algorithm - GREEDY-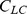
2 (pseudo-code provided in Table 2). We prove the following guarantees for this algorithm.

**Table 2:** Algorithm: GREEDY-*C_LC_*2.

Algorithm: GREEDY-*C_LC_*2	
INPUT: K<N; *G* = (*V*, *E*)	
OUTPUT: V′ ⊆ V, s.t. |*V*′|≤*K* and *V*′ is the greedy solution to Max *C_LC_*.	
1:	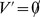
2:	bestVal,curVal = 0
3:	bestInd = null
4:	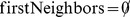
5:	**for** *i*∈*V* **do**
6:	**for** *j*∈*Neighbors*(*i*) **do**
7:	*NW* = *NW*+*j*
8:	**for** *k*∈*Neighbors*(*j*) **do**
9:	**if** *k* ∉ *NW* **then**
10:	*NW* = *NW*+*k*
11:	**end if**
12:	**end for**
13:	**end for**
14:	*N_v_*[*i*] = |*NW*|
15:	**end for**
16:	**for** *i*∈*V* **do**
17:	**for** *j*∈*Neighbors*(*i*) **do**
18:	*N*2*_v_*[*i*] = *N*2*_v_*[*i*]+*N_v_*[*j*]
19:	**end for**
20:	**end for**
21:	lastVal[0..n] = ∞
22:	**while** |*V*′|<*K* **do**
23:	firstNeighbors = firstNeighbors+newNeighbors(firstNeighbors,bestInd) (see note^1^)
24:	*C_LC_*(*V*′) = *C_LC_*(firstNeighbors)
25:	**for** *i*∈*U*−*V*′ **do**
26:	**if** lastVal[i]>bestVal **then**
27:	lastVal[i], curVal = *C_LC_*(*V*′ ∪ {*i*})−*C_LC_*(*V*′)
28:	curVal = *C_LC_*(*V*′ ∪ {*i*})−*C_LC_*(*V*′)
29:	**if** curVal>bestVal **then**
30:	bestVal = curVal
31:	bestInd = i
32:	**end if**
33:	**end if**
34:	**end for**
35:	*V*′ = *V*′∪ {bestInd}
36:	**end while**
37:	RETURN *V*′

1 The function *newNeighbors(V; v)* takes a set of nodes and a new node and adds any neighbors of the new node that are not already in the set.

#### Theorem 7

*Any solution produced by algorithm 2 could also be produced by algorithm 1.*

#### Theorem 8

*GREEDY-* 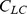 *2 takes* 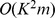 *time.*

In this improved approach, our first intuition was to pre-compute the quantity 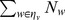 for each node 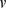 and store it in a data-structure. Next we decided to keep track of all the first neighbors of the set we are building, which allows the algorithm to avoid recalculating that set each loop. This yields a provable improvement in time complexity by a factor of 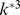. Additionally, we added a practical improvement as well. In a related submodular problem, Leskovec [17] obtained a 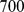 percent increase by “lazy” evaluation of the submodular function (over the basic greedy approach, based on experiments). We include that in this approach by altering line 7, correctly avoiding unnecessary calculations of centrality for poorly-performing nodes. We present experimental evaluations of how this modification affected our problem in the next section.

#### Example 1

*Table 3* *features the improved algorithm selecting a set of three vertices from a small network of 35 primates' relationships. Each column contains a vertex followed by how much that vertex would increase the* 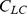 *of the set if it were added to the set. For example, as the algorithm runs through each vertex seeking the first to add to the set, the first vertex is automatically the first greatest increase found, until the fourth vertex is found to generate a higher* 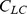 *value, and last in the column is vertex 16, which is then becomes first vertex in the set. In the second and third columns the practical improvement of GREEDY-* 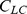 *2 is visible. Each time a* 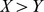 *appears it signifies that a vertex was skipped because in the last iteration it increased* 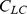 *by less than whatever is the current best increase for this iteration.*

**Table 3:** Example GREEDY-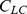
2 Run.

1) 481	1) 160	1) 160
4) 1441	4) 685	2) 160>160
5) 1592	5) 826	3) 160>160
10) 1885	7) 826>703	4) 240
12) 2259	8) 826>279	11) 240>0
13) 2298	9) 826>279	19) 240>69
16) 2727	12) 987	20) 240>0
	18) 987>606	34) 240>80
	23) 987>899	35) 240>80
	24) 987>533	
	25) 987>306	
	26) 987>445	
	27) 987>759	
	28) 987>690	
	29) 987>690	
	30) 987>445	
	31) 987>708	
	32) 987>250	
	33) 987>245	
	34) 987>80	
	35) 987>80	

Each column represents the algorithm choosing a vertex to add to the set; vertices 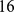, 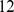, and 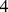 were chosen and in that order. Vertices only appear if they are the maximum addition when considered or if they are ignored (represented by the inequalities). The format is as follows: the vertex considered appears first, followed by a parenthesis, and then either a value or an inequality. The inequality represents that the considered node had a lower addition to the 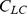 of the set last iteration than the current best addition now, and therefore does not need to be computed this round. A single value represents the addition to 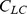 that vertex would contribute.

### Datasets

We examined five different networks in our analysis. They include an a sexual interaction network [18], email network [19], an academic collaboration network [20], a protein interaction network [21], and a social network [22]. Each network is both unweighted and undirected. Our intuition was to utilize networks from a variety of domains in our evaluations.

**Figure 2:**
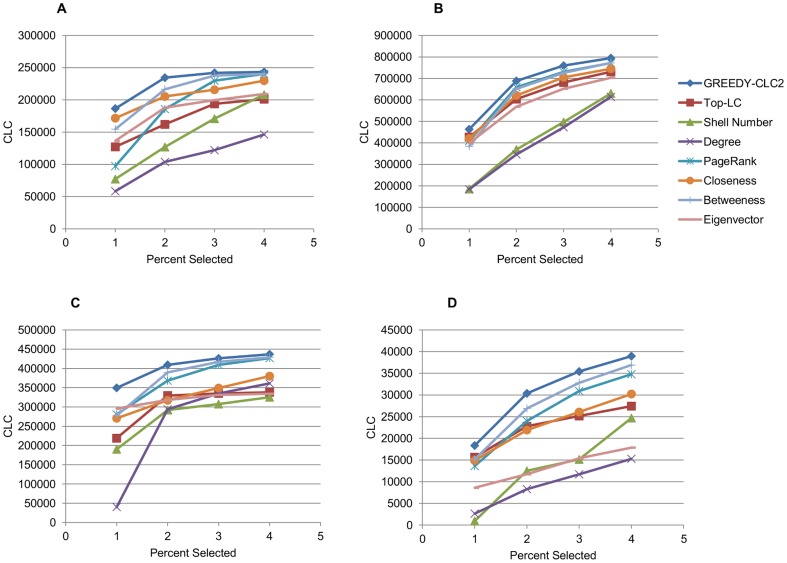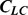 of Sets Chosen with Various Centrality Measures. The 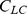 of sets chosen by various centrality measures. Sexual interaction, email, collaboration, and protein interaction networks are respectively graphs A, B, C, and D.

**Figure 3:**
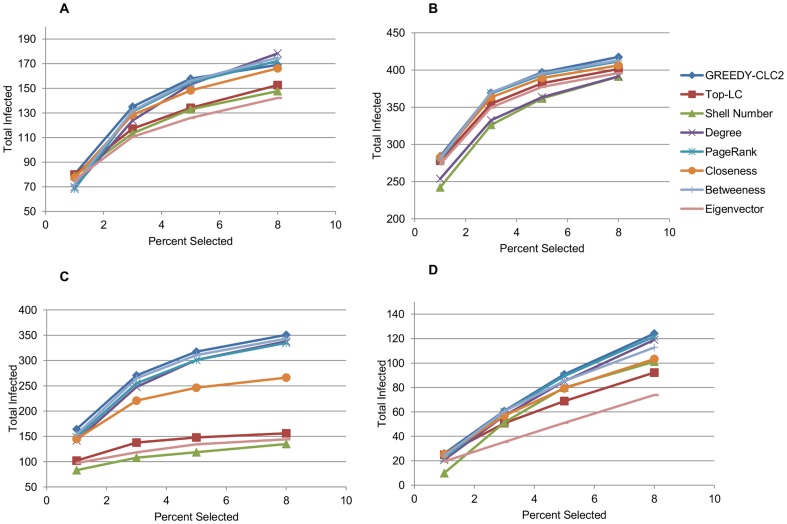
Spreading Impact of Sets Chosen with Various Centrality Measures. The number of vertices infected after 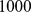 simulation runs to 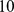 steps in the SIR model, averaged over five subgraphs for each datum. Sexual interaction, email, academic collaboration, and protein interaction networks are respectively graphs A, B, C, and D.

The sexual interaction, email, academic collaboration, and protein interaction networks are denoted A, B, C, and D (respectively) in Figures 2 and 3. We provide some details on these networks in Table 4. The social network was primarily used for run-time analysis (Table 5). These networks are described in more detail below.

**Table 4:** Dataset Information.

Dataset	Nodes	Edges
Brazil Sexual Interaction Network	16730	39044
University Rovira i Virgili Email Network	1133	5451
Academic Collaboration Network	5242	14484
Yeast Protein Interaction Network	1870	2203
Youtube Social Network	13723	76765
Douban Social Network	154907	654188

**Table 5:** Runtime of GREEDY-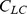 vs GREEDY-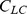
2.

Graph Size	Simple Run Time	Fast Run Time
100	80.37	0.03
200	1502.95	0.19
300	9784.2	0.74
400	35610.68	1.90

Runtimes are in seconds. Each algorithm selected 5% of the given graphs, which are random samples of the email dataset.

The sexual interaction network is an online sex community in Brazil in which a link represents that one of the individuals posted online about a sexual experience with the other individual, resulting in a bipartite graph. The data was extracted from September of 2002 to October of 2008 Luis E. C. Rocha & Holme [18].

The email network is derived from the communications of members of the University Rovira i Virgili. It was extracted in 2003 [19].

The academic collaboration network is derived from the arXiv pre-print server and covers scientific collaborations between authors papers submitted to the General Relativity and Quantum Cosmology category from Jan. 1993–Apr. 2003 [20].

The protein interaction network is a network consisting of protein-protein interactions in yeast [21].

The social network is derived from YouTube, the video-sharing website that allows users to establish friendship links [22]. The sample was extracted in Dec. 2008. Links represent two individuals sharing one or more subscriptions to channels on YouTube.

The Douban network was mined from Douban.com, launched on March 6, 2005, which is a Chinese Web 2.0 website providing user review and recommendation services for movies, books, and music. It is also the largest online Chinese language book, movie and music database and one of the largest online communities in China [23].

### Experimental Set-Up

The runtime experiments on the Douban social media network were conducted on a platform with an Intel X5677 Xeon Processor operating at 3.46 GHz with a 12 MB Cache and 288 GB of physical memory. The machine was running Red Hat Enterprise Linux version 6.1. Only one core was used for experiments. All other experiments were run on a computer equipped with an Intel Core i7 M620 equipped with two cores at 2.67 GHz with 4.00 GB of RAM (only one core was utilized). The machine was running Windows 7. GREEDY-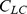 and GREEDY-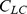
2 were written using Python 2.7.3 in 75 and 80 lines of code, respectively, that leveraged the NetworkX library available from http://networkx.lanl.gov/. The SciPy library from http://www.scipy.org/ was also used for the experimental setup.

We compared our improved algorithm to choosing the top 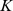 vertices from many common centrality measures. Top-LC refers to choosing the top 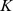 vertices using Local Centrality, rather than trying to optimize Combinatorial Local Centrality. Degree is simply the number of edges a node has. Shell number refers to the greatest core to which a node belongs (see [1] for details). Betweenness measures how many shortest paths, of all vertex pairs in the network, run through a vertex. Closeness is defined as the inverse of farness, where a node's farness is the sum of distances to every other node along shortest paths. Eigenvector centrality and PageRank are recursive measures which take into account both how many neighbors a vertex has and the Eigenvector centrality/Pagerank of those neighbors.

## Results

### Runtime

We first examined the run time of our improved algorithm as opposed to the simple greedy algorithm. Using small subsets of the email network, we prompted each algorithm to select 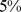 of the subgraph. Table 5 displays the speed-up of the improved algorithm over the simple greedy algorithm even on these very small graphs. The difference is multiple orders of magnitude, aligning with our theoretical results.

Next we wanted to demonstrate that our improved algorithm also performs well with respect to computing other common centrality measures. Taking four of the datasets, the email, sexual interaction, social network, and the Douban network, we generated initial seed sets with GREEDY-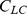
2 and compared this time to how long it took for the NetworkX built in functions for Closeness and Betweenness dictionaries to be calculated, shown in Table 6. Our improved algorithm relies on pre-computation of the value 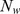, the number of first and second neighbors of each vertex in the graph, so the time it takes to calculate 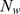 is also included in Table 6. Once the dictionaries for Closeness and Betweenness are found, they must be sorted to deliver the top 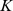 nodes, but that time is negligible next to the time required to build the dictionaries and therefore is not included. The NetworkX implementations for both Closeness and Betweenness are of complexity 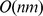 [24], [25]. Recall that the time complexity of GREEDY-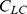
2 is 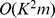, therefore when 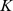 is relatively small compared to 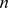 we should expect GREEDY-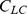
2 to outperform Closeness and Betweenness.

**Table 6:** Runtime of GREEDY-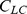
2, Closeness, and Betweenness.

Nodes	Edges	CLC Size	N_w Time	CLC Time	Betweenness	Closeness
1133	5451	249	0.61	2.39	8.64	2.60
16730	39044	1000	16.58	383.89	2307.14	561.54
13723	76765	1000	66.13	407.93	2525.92	693.55
154907	654188	2000	249.22	7129.11	>24 hrs	>24 hrs

Runtimes are in seconds. 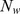 must be precomputed and stored once before GREEDY-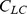
2 can be run.

**Figure 4:**
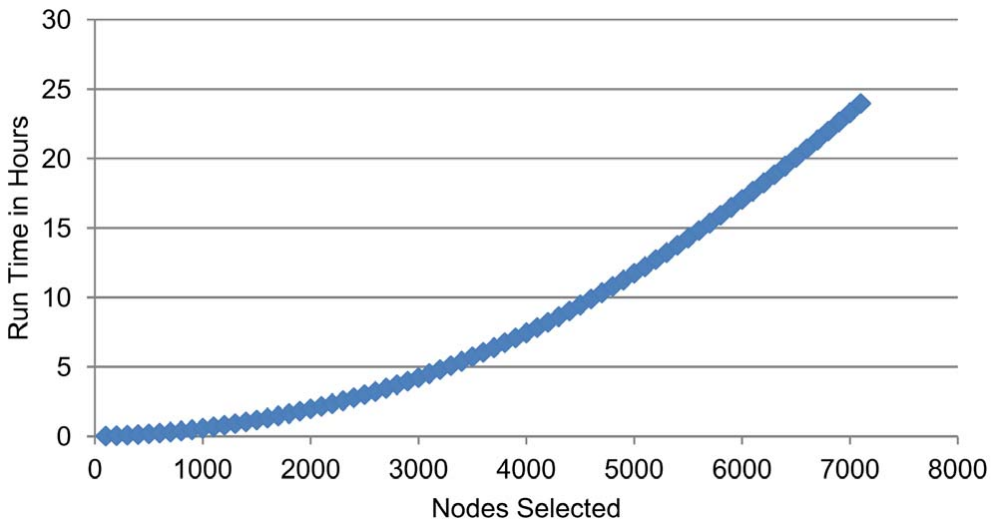
Run Time of GREEDY-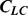
2 on the Douban Social Network. The run time in hours for GREEDY-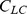
2 to build sets between 100 and 7100 nodes.

Finally, we demonstrated that our GREEDY-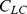
2 algorithm could also deliver results on a larger datasets - which is a more typical need in practical applications dealing with social media site. Here we used a social network extracted from the Douban social media site [23], which consisted of 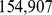 nodes and 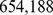 edges. For this experiment, we evaluated the runtime of our algorithm as a function of the cardinality of the solution (Figure 4). We found that a quadratic relationship was maintained (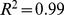) which reflects our complexity result of Theorem 8. Finding a set of 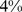 of the population (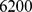 nodes) took 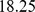 hours, which significantly outperformed other measures. Currently, we are exploring means to further scale this approach, including additional heuristic approximations and parallelization.

### 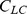 Optimization

To test the efficacy of GREEDY-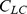
2, we examined five different 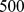 node subgraphs of four separate networks. On each subgraph, we chose the top 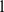, 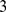, 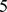, and 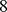 percent of vertices based on several common centrality measures and using GREEDY-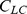
2. First we needed to demonstrate that GREEDY-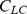
2 does in fact optimize 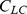 better than other measures. This is difficult to show definitively, because we do not have other algorithms which aim to maximize 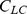 to use as a comparison, but the contrast with common centrality measures is still helpful. In Figure 2 we present the averages of the 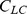 value over those five subgraphs for the subsets chosen by GREEDY-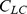
2 versus each of the subsets chosen by selecting the top X percent of nodes using other centrality measures. Figure 2 shows both that sets that have a high 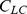 are in practice very different from other measures (i.e. we did not develop a trivially new definition), and then that seeking sets with other centrality measures is not good shortcut to finding sets that have a high 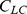. In all cases, GREEDY-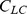
2 chose the set with the highest 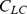, and was an average of 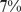 greater than the top performer for each percent and data set pair. On every dataset an analysis of variance (ANOVA) reveals that there is a significant difference in the performance among our algorithm and the centrality measures with respect to increase or decrease in 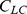 (p-value less than 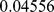 calculated with R version 3.01) except academic collaboration network, which had a p-value between 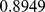 and 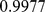 for each percentage trial. Some of the uncertainty in the statistical analysis is attributable to the variance between the random subgraphs, as in many cases average 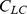 values across all centrality measures differed between two subgraphs as much as 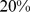.

In some trials, particularly in sexual interaction and academic collaboration (A and C in Figure 2), GREEDY-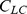
2 reached a maximum 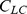 value before selecting 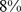 of the graph, at which point the averages of other centrality measures begin to approach GREEDY-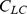
2. However, as 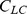 has already been maximized in this case (because the first neighbors of the seed set cover the entire graph), they will never surpass the 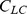 of the smaller set. In a real world scenario, this may be taken advantage of as a way to save advertising costs or focus on a smaller set of the population for epidemic evaluation.

### Epidemic Evaluation

Next the same sets as chosen in the previous section were the initial infectees for 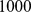 simulation runs over the SIR model. In this paper, to remain consistent with the work of [7], we mimicked their experimental model. After setting our initial infectees to the infected state, we run the SIR model for ten time steps and then sum the recovered and infected vertices to determine the total number of infected vertices. The results, again averaged over the five subgraphs from each network, are shown in Figure 3. The sets chosen by GREEDY-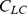
2 spread on average to 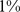 more vertices than the maximum spreader from the rest of the centrality measures over each percent and dataset pair. Furthermore, although occasionally another centrality measure will outperform GREEDY-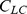
2 on a single cardinality and dataset pair, which measure does so is highly inconsistent. Particularly visible in the sexual interaction network (panel A of Figure 3), GREEDY-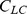
2 did not produce a set as big as 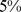 or 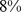 of the graph on every subgraph, so other centrality measures gained an advantage in that they began with more infectees. Interestingly though, 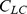 still remained in the top half of the centrality measures, suggesting again a certain threshold after which it is inefficient to continue seeding a graph and a way to conserve real world resources. An analysis of variance (ANOVA) on every dataset reveals that there is a significant difference in the performance among sets chosen by our algorithm and the other centrality measures with respect to increase or decrease in total vertices infected (p-value less than 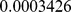 calculated with R version 3.01), except the sexual interaction which had a p-value between 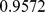 and 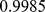 for each percentage trial. However, we also note that this may be a somewhat degenerate case as this particular sexual interaction network consisted of only heterosexual interactions - which leads to a bipartite structure. This may account for the 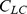 measure covering the entire network without using all of the resources - which in turn led to inconsistent performance against the centrality measures in the simulation trials.

## Discussion

In this paper, we explored the problem of identifying a set of nodes that will cause an epidemic to spread under the SIR model of [7]. To do so, we extended the centrality measure of [7] for sets rather than individual nodes. Though we found that finding a set of nodes that maximizes this combinatorial centrality measurement is NP-hard, we develop a polynomial-time heuristic that we prove to provide the best approximation ratio unless P = NP. We then further improve the performance, both theoretically and practically in a modified version of the algorithm that provides the same theoretical guarantee. We implemented our algorithms and evaluated them on real-world datasets in terms of runtime, ability to maximize the combinatorial centrality measure, and the ability to find sets of nodes that encourage spreading in the SIR model. We found our algorithms to outperform standard approaches in all of these evaluations. Further, we show our approach to scale to networks of 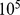 nodes.

Future work could include a modified version of 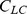 which produces a disease spread mitigation strategy. In such a scenario, we would attempt to find nodes that, if “inoculated” would minimize the maximum value for 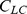 with respect to a given cardinality constraint. Additionally, further evaluation of 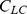 based on different diffusion models, such as those raised in the related work section, is another important direction for further research. In particular, an evaluation of the metric under a classic SIR Model, rather than the variant described in this paper and in [9], would be a good first step.

## Appendix

### Proof of Theorem 1

The Max 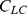 Problem is NP-Hard.

*Proof.*

#### Definition 3

*Max K-Cover* [*15*]

INPUT: *Universe* 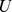, *a set of subsets* 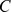, *and natural number* 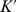


*OUTPUT*: 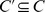, 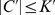 *s.t.* 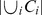 *is maximized*.

Embedding: Given Max K Cover as defined in definition 2.4, we create an instance of the Max 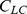 as follows. Form a bipartite graph G by creating a vertex for each 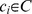 and each 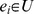. Create a directed edge from 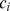 to 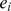 if 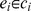. For each vertex corresponding to an element 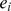 create two additional nodes, 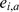 and 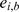. Also add a directed edge from 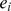 to 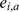 and from 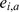 to 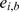. Each node corresponding to a subset 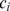 now has a path length of three to some 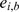.

#### Claim 1

*Embedding of Max K-Cover into Max* 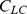 *can be accomplished in polynomial time, as graph G has* 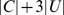 *vertices and* 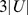 *edges, whose creation takes constant time.*

#### Claim 2

*Given set* 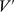 *returned by the an instance of Max* 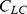 *with* 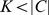 *, the set* 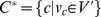 *is the solution to the Max K-Cover problem.*

Suppose by way of contradiction that there exists some set 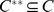 such that 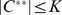 and the number of elements covered by 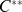 is greater than the number of elements covered by 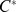. Let 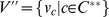.

The number of distinct nearest neighbors for 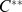 is greater than the number of distinct nearest neighbors of 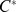. Note that for all vertices corresponding to elements 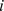, 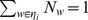 by the construction, and 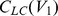 is simply the count of distinct nearest neighbors of set 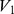. Therefore 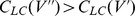, which is a contradiction.

#### Claim 3

*Given set* 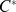 *returned by Max K-Cover, the set* 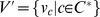 *is a solution to Max* 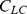 *.*

Suppose by way of contradiction that there exists some 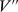 where 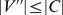 and 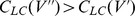. Let 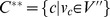.


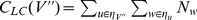
, which under the construction is 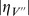. Similarly 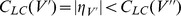. This is equivalent to saying that the number of nearest neighbors covered by set 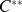 is greater than that of 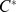, which is a contradiction.

### Proof of Theorem 2

The Dec 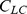 Problem is NP-Complete.

*Proof.* Given an oracle that produces a solution 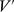, we can clearly check if 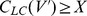 in polynomial time by Theorem 1.

### Proof of Theorem 3

Max 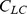 cannot be approximated within 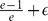 for 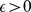 unless P = NP.

*Proof.* Embedding: We use the same embedding as in Theorem 2.1 above.

Let 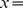 the number of sets covered by some set 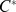 of Max K-Cover.

Let 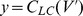 where 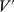 is the set of vertices for Max 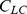.

#### Claim 4


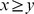
.

Suppose by way of contradiction that 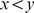. If 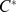 covers fewer neighbors than 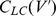 then at least one of those neighbors 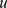 must have a 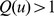. However under the construction all vertices 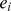 associated with elements have 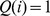 as they each have only one next nearest neighbor 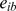 and no neighbors to that vertex, and we have a contradiction.

#### Claim 5


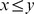
.

Suppose by way of contradiction that 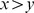. If 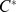 covers more neighbors than 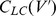 then at least one of those neighbors 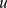 must have a 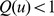. However under the construction all vertices 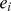 associated with elements have 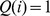 as they each have only one next nearest neighbor 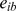 and no neighbors to that vertex, and we have a contradiction.

By the embedding, Claims 1.4 and 1.5, and Thm 4.4 of [15] concerning the limit of approximating set cover, the Max 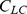 cannot be approximated within 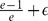 for 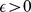 unless P = NP.

### Proof of Lemma 1


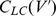
 is monotonic.

*Proof.* Suppose by way of contradiction there exists 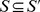 s.t. 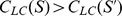. Then


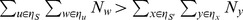
 which implies 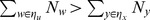.

However, because 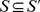 we know 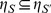 and 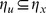.

Because the total neighbors of a subset is necessarily less than the total neighbors of its superset, we have a contradiction.

### Proof of Theorem 4


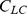
 is sub-modular.

*Proof.* Setup: 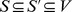; 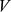 is the set of vertices in graph 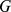; vertex 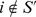;

Suppose by way of contradiction 
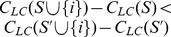
.

Then 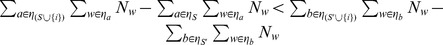


If we let 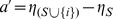 and 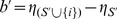 the inequality above becomes:
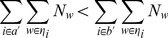
(4)


Note that 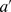 and 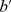 are the sets of neighbors added to sets 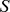 and 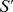, respectively, with the addition of vertex 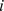.

#### Claim 6


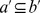


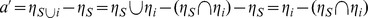



Similarly, 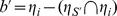. Since 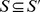, 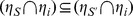, therefore 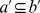.

However, with 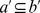, inequality 4 cannot be true, therefore 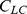 is sub-modular.

### Proof of Theorem 6

GREEDY-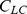 obtains the best approximation ratio unless 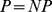.

*Proof.*

#### Claim 7

*GREEDY-* 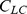 *is a Greedy Algorithm.*

We build set 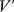 by adding one element at each iteration of the while loop. A new element is chosen by analyzing the increase 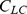 for each node not in 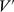 and picking the maximal node. Using a local heuristic to make each choice in a set of decisions is a greedy approach.

#### Claim 8


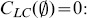
:
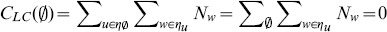



Proof of Theorem: For any monotonic, sub-modular function 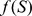 where 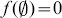, a greedy algorithm guarantees an 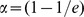 approximation [23]. By Theorems 1 and 4, and Claims 7 and 8, GREEDY-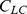 gives an 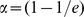 approximation.

By Theorem 2.1 of [2] and the approximation ratio 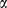 above, 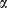 is the best approximation if 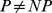.

### Proof of Theorem 5

GREEDY-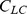 takes 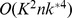 time.

*Proof.*

#### Claim 9


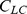
 *takes* 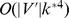


To compute 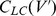, first we iterate through each vertex in 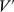. For each vertex, we consider each neighbor, and barring repeated vertices in the set we add those neighbors to a set of first neighbors for set 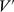, which takes 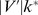. For each vertex in the first neighbor set we count the first and second neighbors, which is no worse than 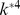. Therefore the time complexity is 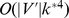.

GREEDY-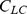 utilizes two looping control structures. The first is a while loop that runs 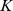 times, and the second is a nested for loop that runs for at most 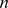 times, for each vertex in the graph. Inside that loop the 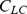 algorithm, 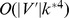, is called twice. The time complexity is then 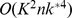.

### Proof of Theorem 7

Any solution produced by algorithm 2 could also be produced by algorithm 1.

*Proof.* Suppose by way of contradiction the condition that lastVal[i]>bestVal caused us to omit the maximal node, 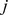, or that the maximal node's last recorded marginal increase in 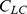 was lower than the current best value. As 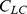 is sub-modular by Thm 2.5, an updated marginal increase of 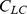 would have to be lower than lastVal[j]. However if the new marginal increase is lower than lastVal[j], it must also be lower than bestVal, and therefore 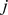 could not be optimal.

### Proof of Theorem 8

*Proof.* GREEDY-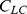
2 takes 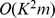 time.

Given that we store 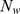 for all vertices and a list 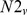 which contains the sum of 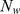 for all neighbors 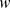 of a node 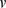, and an alternate form of computing 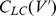 which takes the first neighbors of set 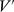, 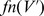:

To compute 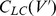, now we simply iterate through the 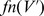 and sum 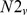 for each, which takes 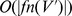. Updating 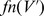 requires adding all new neighbors whenever a new vertex is appended to the set, which takes 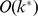 (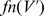 can take multiple vertices, but in the algorithm's implementation it only takes one).

The improved algorithm must also loop until it reaches 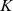 vertices, and considers each vertex in the graph when choosing a new vertex. To choose a new vertex, it must update 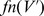 with the potential new neighbors of a possible vertex and calculate 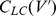, so the complexity is 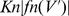. But 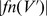 is bound by 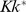 because it is the total number of neighbors of a set of at most 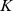 elements, so the complexity may be reduced to 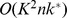. Finally we simplify the factors 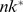 to 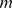.
